# A helping hand: XND1–DOF4.6 interaction modulates Arabidopsis drought tolerance

**DOI:** 10.1093/plcell/koaf129

**Published:** 2025-05-25

**Authors:** Leonard Blaschek

**Affiliations:** Assistant Features Editor, The Plant Cell, American Society of Plant Biologists; Department of Plant & Environmental Sciences, University of Copenhagen, Frederiksberg C 1871, Denmark

Xylem differentiation is strictly regulated. The specialized roles of xylem tracheary elements (TEs) and fibers in long-distance water transport and structural support, respectively, make the precise regulation of their formation pivotal for plant fitness. Xylem differentiation is orchestrated by a group of NAC domain–containing transcription factors (TFs), specifically Vascular NAC-Domain (VND) and NAC Secondary Wall Thickening Promoting Factor (NST) proteins, forming functional homo and hetero dimers. These TFs induce the transcription of genes directing secondary cell wall biosynthesis, lignification, and programmed cell death. Expression of NSTs and VNDs is restricted to future xylem TEs and fibers through cell type– and organ-specific repression by TFs such as WRKY15, WRKY12, and ZFP2 (Wang et al. 2010; Ge et al. 2020; Low et al. 2025). The timeline of differentiation is further fine-tuned post transcriptionally by Xylem NAC Domain 1 (XND1). XND1 heterodimerizes with VNDs and NSTs, attenuating their capacity for transcriptional activation and/or sequestering them into the cytosol (Zhang et al. 2020; Zhong et al. 2021). Loss of XND1 accelerates xylem differentiation, leading to shorter TEs and dwarfed plants without affecting total TE number (Zhong et al. 2021).

Now, **Bingli Ding and colleagues (Ding et al. 2025)** reveal another piece of the regulatory puzzle surrounding xylem differentiation. They show that DOF4.6, previously identified as a negative regulator of TE formation (Ramachandran et al. 2020), acts in concert with XND1 to fine-tune vascular capacity in roots. The *dof4.6* loss-of-function mutant showed accelerated TE differentiation in roots, coinciding with higher root hydraulic conductivity. These phenotypes were nearly identical to those of the *xnd1* loss-of-function mutant. Moreover, *dof4.6 xnd1* double mutants revealed an epistatic relationship between the 2 genes, suggesting that they act in the same pathway. Accordingly, it turns out that DOF4.6 and XND1 interact with each other directly, as confirmed with GST pull-down, split luciferase, bimolecular fluorescence complementation, and co-immunopurification experiments. This interaction increased the capacity of XND1 to bind to the promoter of *XCP1*, a protease directing TE programmed cell death at the end of their differentiation. While the effects on TE differentiation were virtually identical between *dof4.6* and *xnd1* mutants, the former showed a higher resistance to osmotic stress. This suggested additional, *XND1*-independent functions of DOF4.6 in the regulation of water transport. Indeed, the authors found that expression of aquaporins *PIP2;5* and *PIP2;6* was negatively regulated by DOF4.6 but not XND1. In addition, the accelerated TE formation and transcriptional changes in *dof4.6* mutant plants improved their drought resistance. These results suggest a model in which DOF4.6 and XND1 act in concert to control axial water transport by delineating TE differentiation, while DOF4.6 additionally regulates aquaporins to modulate lateral water transport (Figure).

**Figure.**
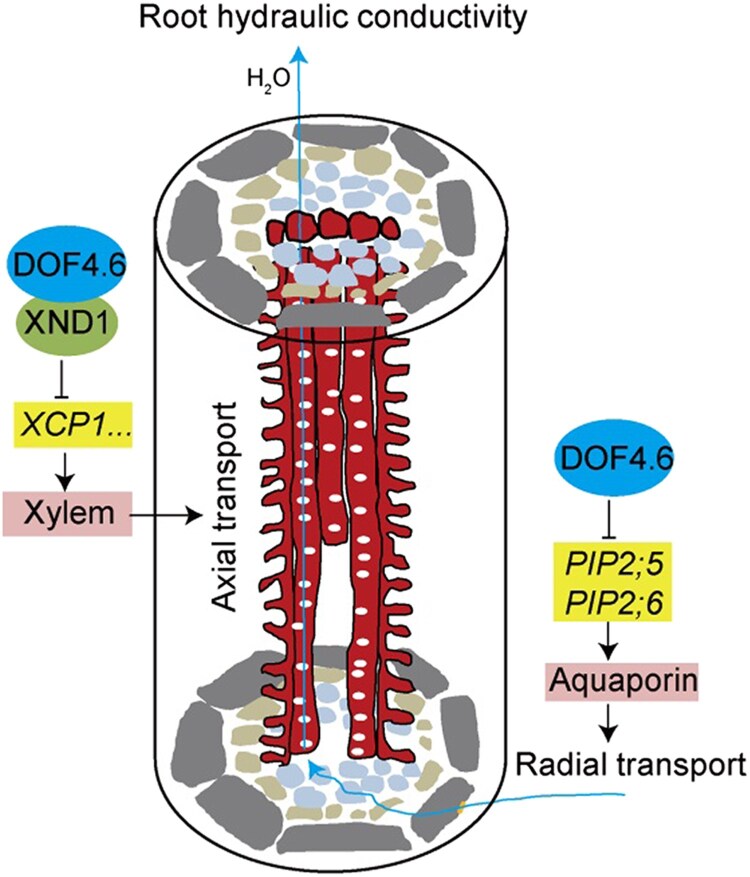
Schematic representation showing the effects of DOF4.6 on root water transport. Reprinted from Ding et al. (2025), Figure 9.

Previously, XND1 was believed to affect xylem differentiation primarily through post-transcriptional repression of VNDs and NSTs. Ding et al. (2025) show that XND1, in complex with DOF4.6, can also directly repress genes downstream of VNDs, such as XCP1. How exactly the direct transcriptional repression by XND1 and DOF4.6 interacts with NST/VND sequestration by XND1 is still hard to gauge. Additionally, it remains to be shown how these effects vary between organs and developmental stages. The regulatory circuitry of xylem differentiation is dynamic and tissue specific, as highlighted by varying roles of DOF4.6 in shoots compared with roots (Ramachandran et al. 2020) and potentially inverted transcriptional responses of aquaporins in 10-day-old and 21-day-old *dof4.6* seedlings (Ding et al. 2025). Lastly, how DOF4.6 might affect the reported positive effect of XND1 on biotic resistance (Tang et al. 2018) is yet to be investigated. The many open questions notwithstanding, the work by Ding and colleagues brings us a step closer to fully appreciating the sophisticated regulatory network underpinning the plant vasculature.

## Recent related articles in *The Plant Cell*:

- Liu et al. (2025) profiled the miRNome during TE differentiation in Arabidopsis and identified miRNA coexpression networks functioning at the different stages of TE differentiation.
- Pfaff et al. (2024) introduced a method to induce tracheary element transdifferentiation of isolated protoplasts that can be used to study patterned secondary cell wall biosynthesis in a tissue-free environment and various mutant backgrounds.

## Data Availability

No original data was generated for this *In Brief*.

## Dive Curated Terms

The following phenotypic, genotypic, and functional terms are of significance to the work described in this paper:

WRKY15 Gramene: AT2G23320
WRKY15 Araport: AT2G23320

ZFP2 Gramene: AT5G57520
ZFP2 Araport: AT5G57520

xylem AmiGo: PO:0005352

XND1 Gramene: AT5G64530
XND1 Araport: AT5G64530

XCP1 Gramene: AT4G35350
XCP1 Araport: AT4G35350

XCP2 Gramene: AT1G20850
XCP2 Araport: AT1G20850
